# IL-1α promotes liver inflammation and necrosis during blood-stage *Plasmodium chabaudi* malaria

**DOI:** 10.1038/s41598-019-44125-2

**Published:** 2019-05-20

**Authors:** Maria Nogueira de Menezes, Érika Machado Salles, Flávia Vieira, Eduardo Pinheiro Amaral, Vanessa Zuzarte-Luís, Alexandra Cassado, Sabrina Epiphanio, José Maria Alvarez, José Carlos Alves-Filho, Maria Manuel Mota, Maria Regina D’Império-Lima

**Affiliations:** 1 Instituto de Ciências Biomédicas, Universidade de São Paulo, São Paulo, Brazil; 2 Instituto de Medicina Molecular, Faculdade de Medicina, Universidade de Lisboa, Lisboa, Portugal; 3 Faculdade de Ciências Farmacêuticas, Universidade de São Paulo, São Paulo, Brazil; 4 Escola de Medicina de Ribeirão Preto, Universidade de São Paulo, Ribeirão Preto, Brazil

**Keywords:** Interleukins, Inflammation, Malaria

## Abstract

Malaria causes hepatic inflammation and damage, which contribute to disease severity. The pro-inflammatory cytokine interleukin (IL)-1α is released by non-hematopoietic or hematopoietic cells during liver injury. This study established the role of IL-1α in the liver pathology caused by blood-stage *P. chabaudi* malaria. During acute infection, hepatic inflammation and necrosis were accompanied by NLRP3 inflammasome-independent IL-1α production. Systemically, IL-1α deficiency attenuated weight loss and hypothermia but had minor effects on parasitemia control. In the liver, the absence of IL-1α reduced the number of TUNEL^+^ cells and necrotic lesions. This finding was associated with a lower inflammatory response, including TNF-α production. The main source of IL-1α in the liver of infected mice was inflammatory cells, particularly neutrophils. The implication of IL-1α in liver inflammation and necrosis caused by *P. chabaudi* infection, as well as in weight loss and hypothermia, opens up new perspectives for improving malaria outcomes by inhibiting IL-1 signaling.

## Introduction

Hepatosplenomegaly is a hallmark of malaria, which is a parasitic disease caused mainly by *Plasmodium falciparum* and *Plasmodium vivax* that results in almost half a million deaths per year^1^. The severe forms of the disease affect several tissues and organs, even when the most marked clinical manifestations involve a single organ^2^. Hepatic failure symptoms, such as jaundice and high serum levels of alanine transaminase (ALT) and aspartate transaminase (AST), are frequently observed in malaria patients^3–5^. The hepatic tissue from *P. falciparum*-infected patients with acute liver failure shows necrotic lesions and hyperplastic Kupffer cells loaded with the malarial pigment, named hemozoin^3^. *P. vivax* infection also causes hepatic dysfunction^5^. Hyperbilirubinemia (serum bilirubin > 3 mg/dL), together with a more than 3-fold increase in serum aminotransferases levels, characterizes malarial hepatopathy^4,6^, which is associated with other complications, such as cerebral malaria, shock and acute kidney injury^6^. Therefore, it is proposed that malaria-induced acute liver failure is involved in the pathogenesis of cerebral malaria^7^.

Hepatic necrosis, leukocyte infiltration and the increased production of pro-inflammatory cytokines in the liver influence the outcome of murine malaria caused by blood-stage infections with *Plasmodium yoelli, Plasmodium chabaudi* and *Plasmodium berghei*^8–11^. Intravascular hemolysis, resulting from *Plasmodium* infection, is implicated in the liver damage through the oxidative stress induced by the free heme overload^12^. Hepatic inflammation is triggered in response to infection and tissue injury and is characterized by the recruitment of innate immune cells and the production of pro-inflammatory cytokines, such as tumor necrosis factor alpha (TNF-α), interleukin (IL)-1 and IL-6^13^. This inflammatory environment protects the host from infection, but it can also contribute to exacerbating the hepatic tissue destruction.

IL-1α and IL-1β are important cytokines of the IL-1 family that induce fever, neutrophil influx and activation, monocyte recruitment, prostaglandin synthesis, T- and B-cell activation and cytokine production^14–16^. These cytokines share the same receptor (IL-1R) but are different in the manner in which they are produced, released and exert their function. IL-1α is constitutively expressed as a biologically active cytokine inside different cell types and does not require additional processing for IL-1R signaling^17^. Therefore, necrotic cells passively release IL-1α, which leads to the rapid recruitment of inflammatory cells^18,19^. Due to these characteristics, IL-1α is considered a damage-associated molecular pattern (DAMP), since it acts as an alarm signal to initiate inflammation in response to tissue injury^20^. IL-1α signaling in hepatocytes amplifies the inflammatory response and exacerbates liver damage^21^, but the source of this IL-1α is still in controversy. It is known that Kupffer cells and hepatocytes produce IL-1α that promotes inflammatory response induced by liver damage^18,19,22^. Moreover, the IL-1α released during hypoxia initiates sterile inflammation by inducing the early recruitment of IL-1α-producing neutrophils, whereas IL-1β promotes the late recruitment and retention of IL-1β-producing macrophages^15^. In some circumstances, the NLRP3 inflammasome activation promotes IL-1α release by macrophages and dendritic cells. While soluble NLRP3 inflammasome agonists, such as nigericin and ATP, induce an inflammasome-dependent release of IL-1α, particulate stimuli, such as alum, silica and monosodium urate crystals (MSU), promote inflammasome-independent IL-1α release^23^.

A high incidence of the *IL1A* gene polymorphism (−4845G > T), which leads to an increased IL-1α production, is described in Vietnamese patients with severe malaria, and high serum levels of IL-1β are also associated with severe disease in individuals from West Africa^24,25^. In murine malaria, IL-1 synergizes with TNF-α to promote nitric oxide production and hypoglycemia^26^. Additionally, the up-regulation of IL-1α in the kidneys promotes the development of glomerulonephritis in *Plasmodium*-infected mice^27^. Pure synthetic hemozoin induces the production of both IL-1α and IL-1β in a systemic and local manner when injected in the mouse peritoneal cavity^28^. However, the importance of IL-1α in the liver pathology caused by *Plasmodium* infection has not been thoroughly elucidated to date. In this study, we address this issue and demonstrate the essential role of IL-1α in liver injury and inflammation during acute malaria and the source of this cytokine in this context.

## Results

### Acute *P. chabaudi* malaria causes liver necrosis and inflammation concomitantly with IL-1α production

**Figure 1:**
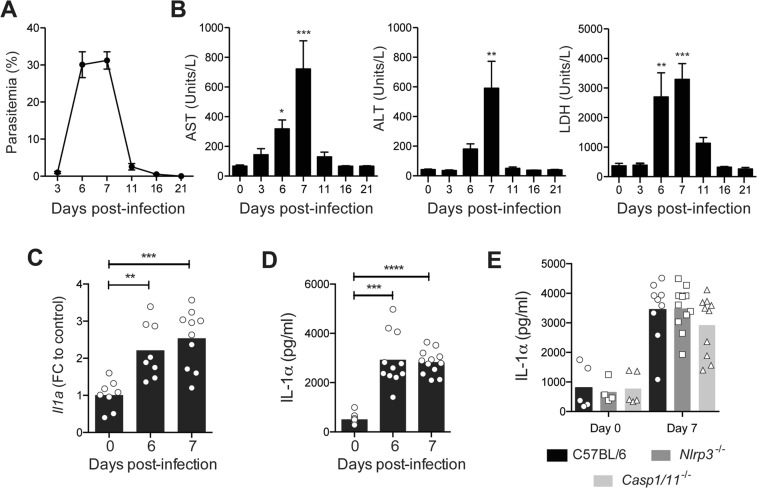
Serum levels of damage-associated enzymes increases concomitantly with IL-1α production in the liver during *P. chabaudi* malaria. Mice were infected with 1 × 10^6^ *P. chabaudi*-iRBCs. Non-infected mice (day 0) were used as controls. (**A,B**) The parasitemia curve and the serum AST, ALT and LDH concentrations in C57BL/6 mice. The data are expressed as the means ± SD (*n* = 3) of one representative experiment out of three. (**C**) *Il1a* mRNA levels in the liver tissue of C57BL/6 mice. FC, fold change. (**D**) IL-1α levels in the liver cell supernatants from C57BL/6 mice. (**E**) IL-1α levels in the liver cell supernatants from C57BL/6, *Nlrp3*^−/−^ and *Casp1/11*^−/−^ mice. (**C**–**E**) The data were pooled from three independent experiments (*n* = 4–12). Significant differences were observed between non-infected and infected mouse groups with *p < 0.05, **p < 0.01, ***p < 0.001 and ****p < 0.0001, using the Kruskal-Wallis multiple comparison test.

To verify the production of IL-1α during hepatic necrosis and inflammation induced by blood-stage malaria, we first evaluated the serum concentration of the liver damage indicators enzymes AST, ALT and LDH. In parallel with the parasitemia (Fig. 1A), enzymes levels increased and reached their maximum levels at days 6 and 7 p.i., decreasing thereafter (Fig. 1B). Because IL-1α and IL-1β cytokine production is associated with tissue damage and inflammation^29^, we assessed these cytokines in the liver of *P. chabaudi*-infected C57BL/6 mice at days 6 and 7 p.i. The *Il1a* mRNA expression was up-regulated in the hepatic tissue (Fig. 1C), and IL-1α protein levels increased in the liver homogenates (Fig. 1D). Showing the independence of the NLRP3 inflammasome for IL-1α production, a similar concentration was observed in infected C57BL/6, *Nlrp3*^−/−^ and *Casp1/11*^−/−^ mice (Fig. 1E). Of note, the IL-1β remained at basal levels in the liver homogenates at days 6 and 7 p.i. (Supplementary Fig. 1). Therefore, *P. chabaudi* malaria induces NLRP3 inflammasome-independent IL-1α production in the liver.

**Figure 2:**
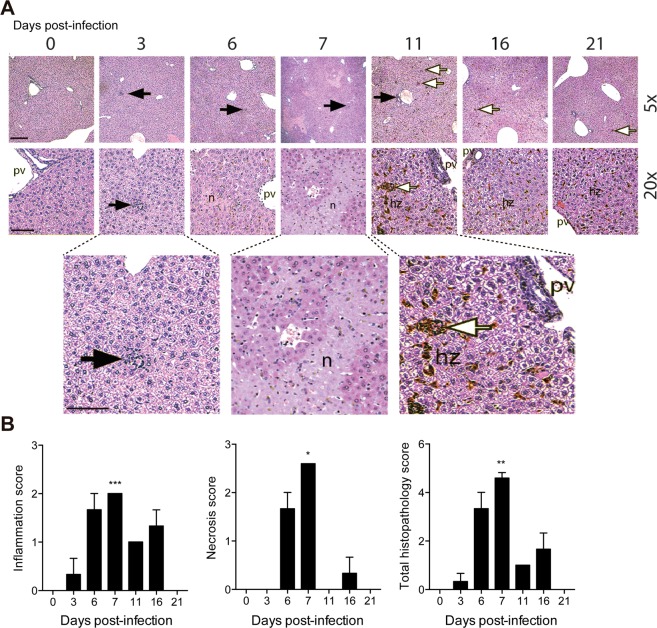
IL-1α production is associated with liver inflammation and necrosis caused by acute *P. chabaudi* malaria. C57BL/6 mice were infected with 1 × 10^6^ *P. chabaudi*-iRBCs. Non-infected mice (day 0) were used as controls. (**A**) H&E-stained liver sections showing necrotic foci (n), cellular infiltration (black arrows), extramedullary hematopoiesis (white arrows), hemozoin pigments (hz) and portal vein (pv). Original magnification is 5x (bar scale corresponds to 300 μm) and 20x (scale bar corresponds to 50 μm). (**B**) Semiquantitative histopathological scores of inflammation, necrosis and total histopathology (inflammation and necrosis). The data are expressed as the means ± SD (*n* = 3) of one representative experiment out of two. Significant differences were observed between non-infected and infected mouse groups with *p < 0.05, **p < 0.01 and ***p < 0.001, using the Kruskal-Wallis test multiple comparison test.

Consistent with the hepatic enzyme kinetics, many inflammatory and necrotic foci were observed in the liver tissue during the acute infection (Fig. 2A). Extramedullary hematopoiesis was observed, mainly after parasitemia control, when the accumulation of the hemozoin pigments became evident. The histopathological analysis showed that the peak of the hepatic tissue damage was at day 7 p.i., considering the necrosis, inflammation and total histopathology scores (Fig. 2B). These data show that acute *P. chabaudi* malaria induces IL-1α production in the liver during hepatic necrosis and inflammation.

### IL-1α deficiency leads to higher parasitemia but attenuates the body weight loss, hypothermia and liver damage

**Figure 3:**
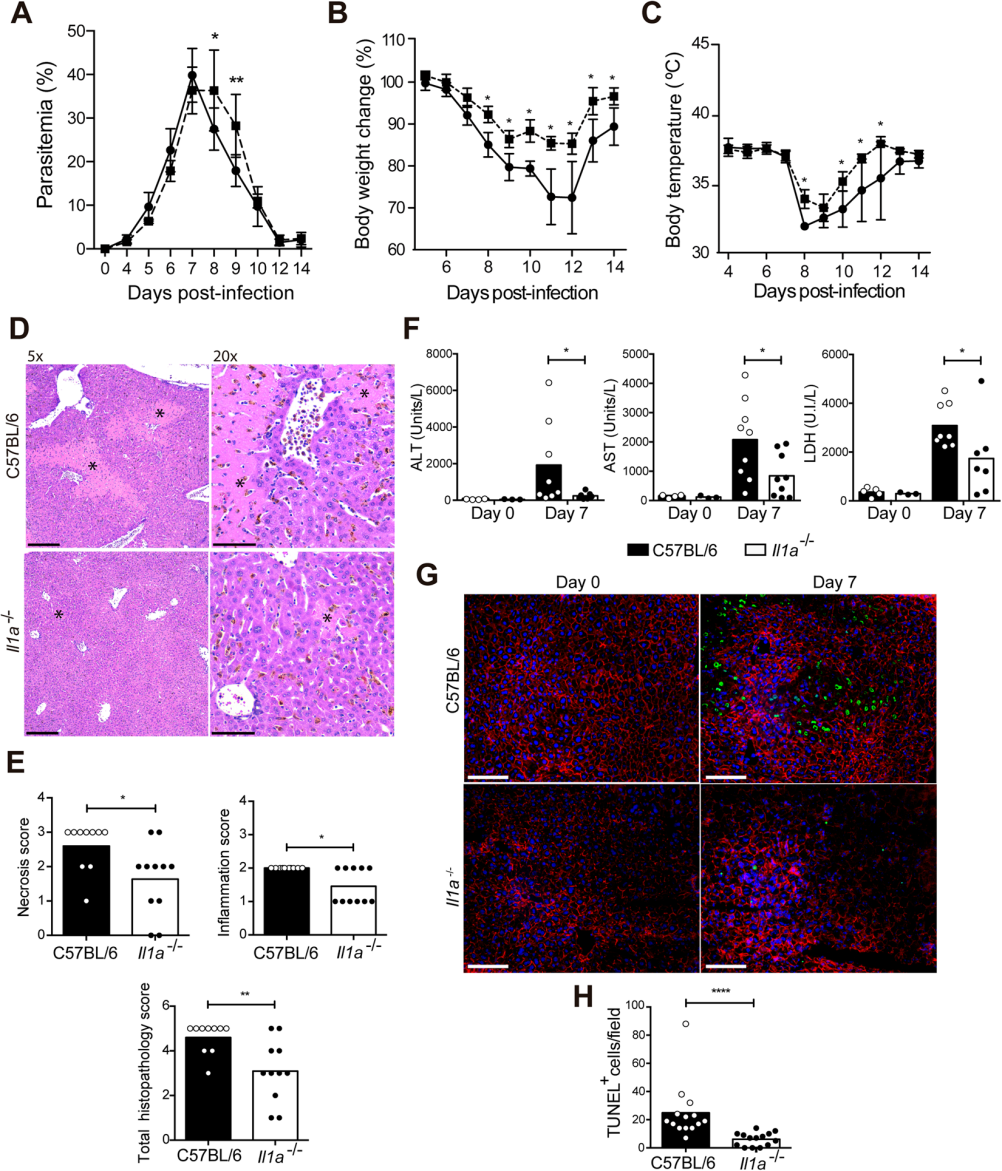
IL-1α deficiency leads to transient higher parasitemia but attenuates the body weight loss, hypothermia and liver damage. C57BL/6 and *Il1a*^−/−^ mice were infected with 1 × 10^6 ^*P. chabaudi*-iRBCs. Non-infected mice (day 0) were used as controls. (**A**) Parasitemia curves. (**B**) Body weight change. (**C**) Body temperature. (**A**–**C**) The data are expressed as the means ± SD (*n* = 5) of one representative experiment out of three. C57BL/6 (solid line) and *Il1a*^−/−^ (dashed line). (**D**) H&E-stained liver sections showing necrotic foci (asterisk) and cellular infiltration at day 7 p.i. (5x and 20x magnification; bar scale corresponds to 300 and 50 μm, respectively). (**E**) Semiquantitative histopathological scores of inflammation, necrosis and total histopathology (inflammation and necrosis) at day 7 p.i. The data were pooled from three independent experiments (*n* = 10–11). (**F**) Serum ALT, AST and LDH concentrations. The data were pooled from three independent experiments (*n* = 3–9). (**G**) Immunofluorescence staining for TUNEL (green), F-actin (red) and nuclei (DAPI, blue) in the frozen liver sections (40x magnification; bar scale corresponds to 100 μm). (**H**) TUNEL^+^ cells per field in the liver sections. The data are expressed as the means ± SD (*n* = 3) of one representative experiment out of three. Significant differences were observed between C57BL/6 and *Il1a*^−/−^ mouse groups with *p < 0.05, **p < 0.01 and ****p < 0.0001, using the Two-way ANOVA and Sidak’s multiple comparisons test (**A**–**C**) or the Mann-Whitney test (**F**, **E** and **H**).

To investigate the role of IL-1α in the disease outcome, parasitemia, weight loss and hypothermia were compared in infected *Il1a*^−/−^ and C57BL/6 mice after *P. chabaudi* infection. These mice showed similar parasitemias until day 7 p.i., but higher levels were found in *Il1a*^−/−^ mice compared to C57BL/6 mice at days 8 and 9 p.i. (Fig. 3A). Despite this difference, both mouse groups efficiently controlled acute parasitemia at day 12 p.i. Moreover, from day 8 p.i., the loss of body weight and hypothermia was attenuated in *Il1a*^−/−^ mice compared to C57BL/6 mice (Fig. 3B,C). *Il1a*^−/−^ and C57BL/6 mice presented comparable initial body weights (Supplementary Fig. 2), suggesting that the food intake was similar for these mouse strains before infection. In view of this data, we concluded that IL-1α promotes body weight loss and hypothermia but plays a minor role in parasite control.

Concerning the liver, at day 7 p.i., the liver histopathological analysis revealed fewer necrotic lesions in *Il1a*^−/−^ mice compared to C57BL/6 mice (Fig. 3D). The hepatic inflammation and total histopathology scores were also lower in infected *Il1a*^−/−^ mice (Fig. 3E). Furthermore, at 7 days p.i., the ALT, AST and LDH serum levels were reduced in *Il1a*^−/−^ mice compared to C57BL/6 mice (Fig. 3F). Confirming that the cell death processes were attenuated in the absence of IL-1α, lower numbers of TUNEL^+^ cells were found at 7 days p.i. in the liver sections from *Il1a*^−/−^ mice than in those from C57BL/6 mice (Fig. 3G,H). These results show that IL-1α promotes hepatic cell death in the liver during acute *P. chabaudi* malaria.

### *P. chabaudi*-induced liver inflammation is reduced in the absence of IL-1α

**Figure 4:**
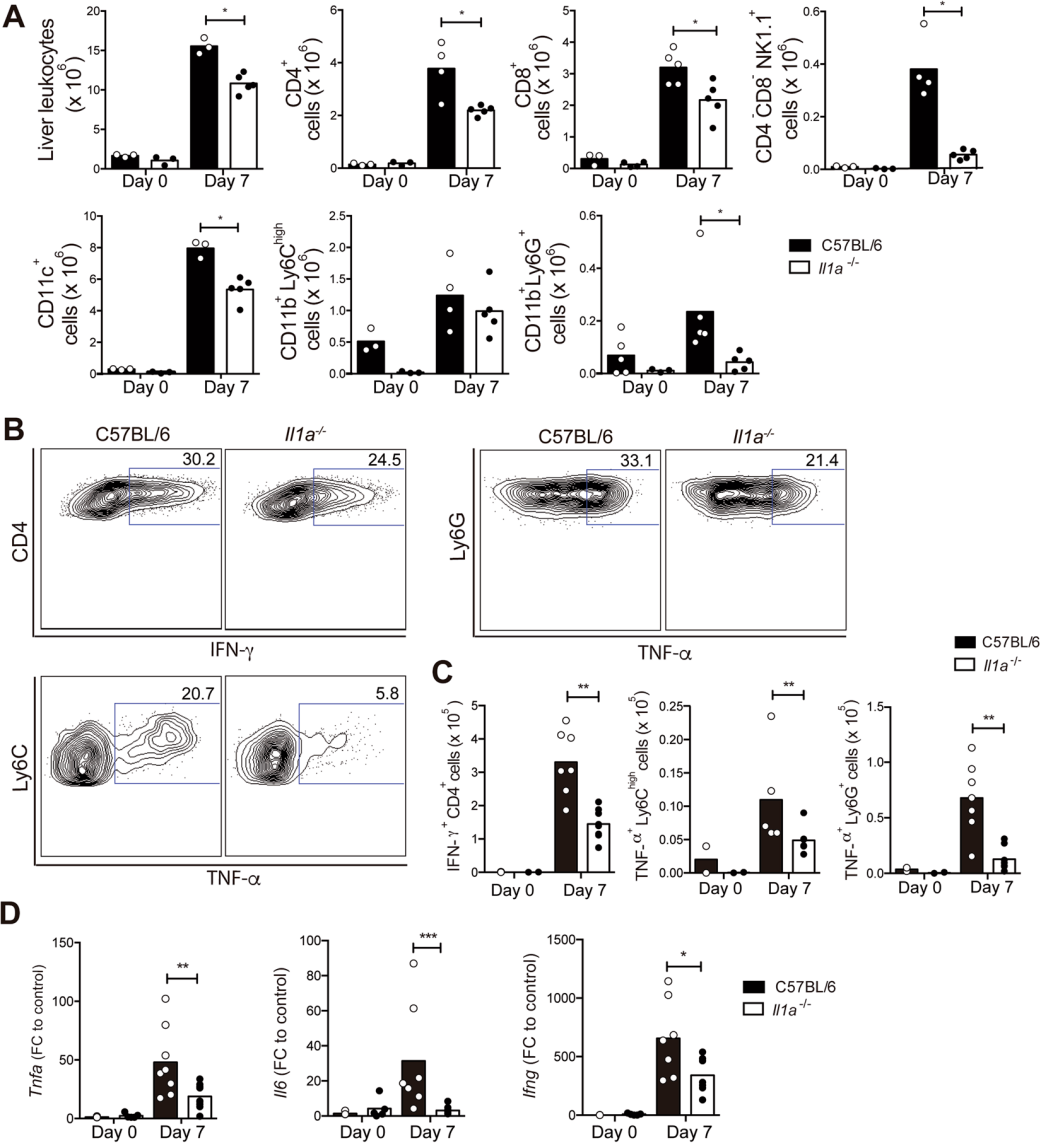
*P. chabaudi*-induced liver inflammation is reduced in the absence of IL-1α. C57BL/6 and *Il1a*^−/−^ mice were analyzed at day 7 p.i. with 1 × 10^6 ^*P. chabaudi*-iRBCs. Non-infected mice (day 0) were used as controls. (**A**) The number of total leukocytes and CD4^+^, CD8^+^, CD4^-^CD8^-^NK1.1^+^, CD11c^+^, CD11b^+^ Ly6C^high^ and CD11b^+^Ly6G^+^ cells in the liver. The data are expressed as the means ± SD (*n* = 3–5) of one representative experiment out of three. (**B**) Contour plots of IFN-γ by CD4^+^ cells and TNF-α production by myeloid cells (CD11b^+^Ly6G^+^ and CD11b^+^Ly6C^high^). (**C**) Numbers of IFN-γ^+^CD4^+^, TNF-α^+^CD11b^+^Ly6C^high^ and TNF-α^+^CD11b^+^Ly6G^+^ cells per liver. (**D**) The relative *Tnfa, Il6* and *Ifng* mRNA expression in the liver tissue. The data were pooled from two independent experiments (*n* = 3–8). Significant differences were observed between C57BL/6 and *Il1a*^−/−^ mouse groups with *p < 0.05, **p < 0.01 and ***p < 0.001, using the Mann-Whitney test.

Evidencing the contribution of IL-1α to *P. chabaudi*-induced hepatic inflammation, lower numbers of leukocytes were harvested from the collagenase-digested livers of infected *Il1a*^−/−^ mice compared to infected C57BL/6 mice (Fig. 4A). The CD4^+^, CD8^+^, NK1.1^+^, CD11c^+^ and CD11b^+^Ly6G^+^ populations were reduced in the absence of IL-1α, whereas similar numbers of CD11b^+^Ly6C^high^ cells were observed in infected *Il1a*^−/−^ and C57BL/6 mice. We also evaluated the spleen, which is a fundamental organ for the innate and adaptive immune response to malaria^30,31^. In contrast to the liver inflammatory response, IL-1α exerted no apparent change in the splenic leukocyte populations (Supplementary Fig. 3A).

It is well-known that pro-inflammatory cytokines, such as TNF-α, IL-6 and IFN-γ, play an important role in both protection and pathology during *Plasmodium* infection^13,32^. TNF-α and IL-6 are also frequently associated with hepatic damage and inflammation^22,33^. Therefore, IFN-γ and TNF-α production, by the liver leukocytes from C57BL/6 and *Il1a*^−/−^ mice, were evaluated at 7 days p.i. by intracellular staining. The CD4^+^IFN-γ^+^ population was significantly reduced in infected *Il1a*^−/−^ mice compared to infected C57BL/6 mice (Fig. 4B,C). Regarding TNF-α production, IL-1α deficiency resulted in a huge decrease both in the frequency and number of TNF-α^+^CD11b^+^Ly6C^high^ and TNF-α^+^CD11b^+^Ly6G^+^ cells per liver (Fig. 4B,C). The mRNA levels of TNF-α, IL-6 and IFN-γ were also lower in the liver of infected *Il1a*^−/−^ mice compared to infected C57BL/6 mice (Fig. 4D). Splenic CD4^+^ T cells are a main source of IFN-γ during acute *P. chabaudi* infection^34^, and this response was not affected by the absence IL-1α (Supplementary Fig. 3B).

All together, these data show that IL-1α promotes leukocyte recruitment and pro-inflammatory cytokine production in the liver during *P. chabaudi* infection, but it has no apparent effect on the splenic cell populations and IFN-γ production by CD4^+^ T cells.

### Inflammatory cells are a major source of IL-1α in acutely *P. chabaudi*-infected mice

**Figure 5:**
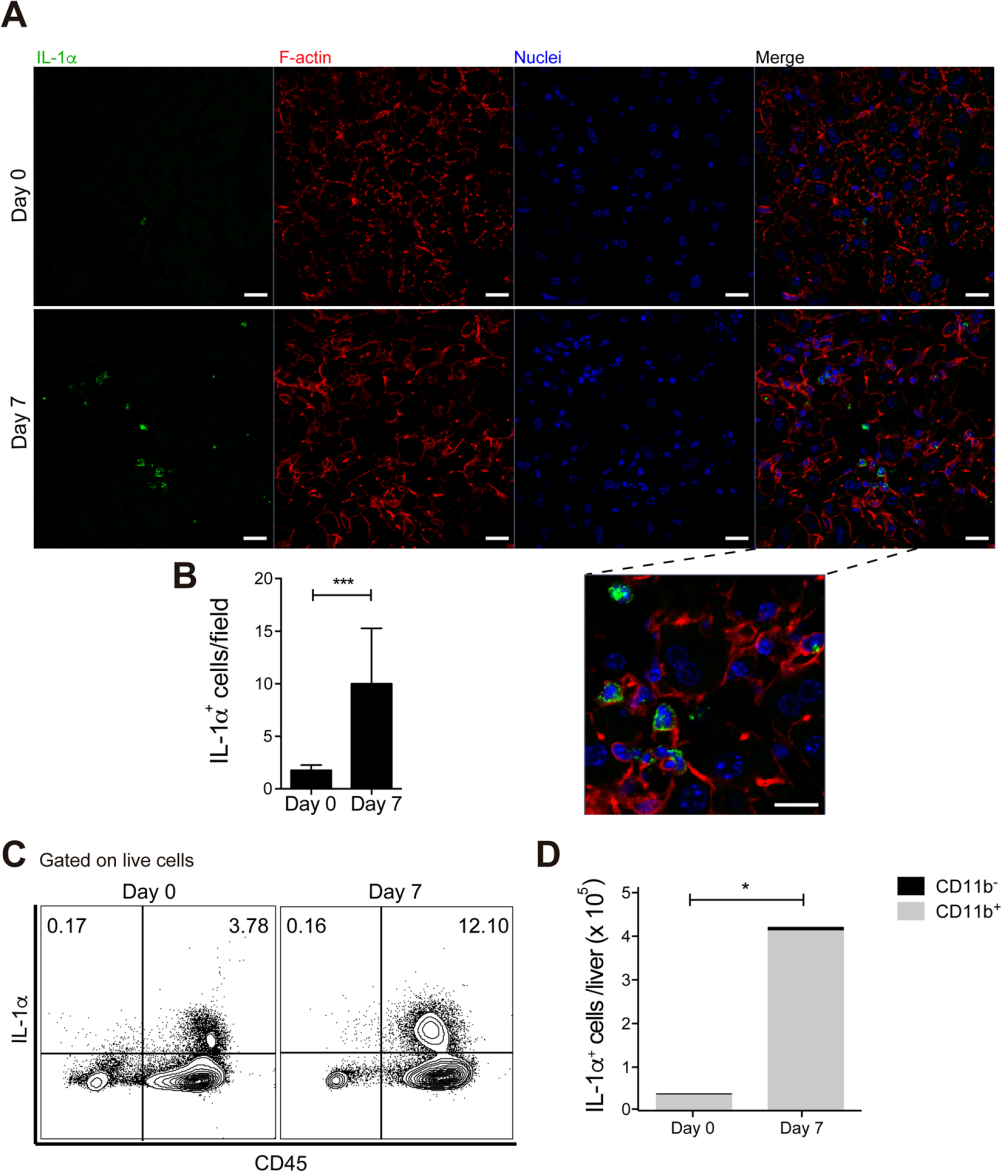
Inflammatory cells are a major source of IL-1α in the liver during *P. chabaudi* malaria. C57BL/6 mice were infected with 1 × 10^6^ *P. chabaudi*-iRBCs. Non-infected mice (day 0) were used as controls. (**A**) Immunofluorescence staining for IL-1α (green), F-actin (red) and nucleus (DAPI, blue) in the liver sections (40x magnification, scale bars correspond to 20 μm). (**B**) Quantification of the number of IL-1α^+^ cells per field in the liver sections. The data are expressed as the means ± SD (*n* = 3–5) of one representative experiment out of two. (**C**) Contour plots of IL-1α production by CD45^−^ and CD45^+^ cells (gated on live cells). (**D**) The number of IL-1α^+^CD45^+^ cells distributed in CD11b^−^ and CD11b^+^ cell populations. The data are expressed as the means ± SD (*n* = 3–5) of one representative experiment out of two. Significant differences were observed between indicated groups with *p < 0.05 and ***p < 0.001, using the Mann-Whitney test.

In order to investigate what is the source of the liver IL-1α that promotes the inflammation and necrosis during acute *P. chabaudi* infection, we performed immunofluorescence analysis of the liver sections of infected C57BL/6 mice. The analysis revealed the presence of IL-1α-producing cells (Fig. 5A), which were in higher numbers compared to non-infected controls (Fig. 5B). The enlarged image of these cells suggested that they were leukocytes, considering the morphology and the reduced size of their nuclei in comparison to those of hepatocytes. Flow cytometry analysis of liver cells confirmed that IL-1α producing cells were positive for the leukocyte marker CD45 and that they were in higher frequency and number at day 7 p.i. in comparison to non-infected mice (Fig. 5C,D). Moreover, the great majority of the IL-1α^+^CD45^+^ population also expressed CD11b (Fig. 5D).

**Figure 6:**
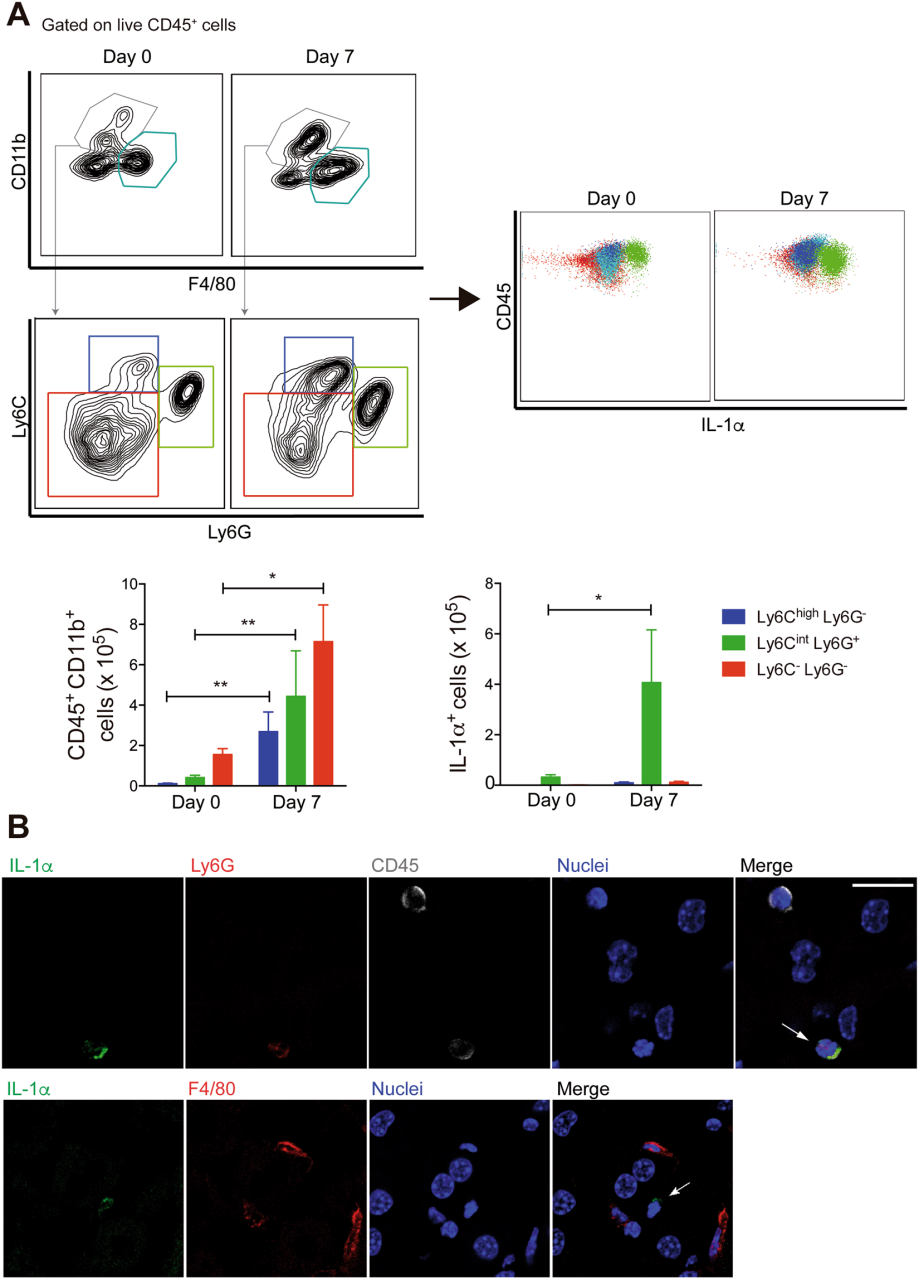
Neutrophils are a major source of IL-1α in the liver during *P. chabaudi* malaria. C57BL/6 mice were analyzed at day 7 p.i. with 1 × 10^6 ^*P. chabaudi*-iRBCs. Non-infected mice (day 0) were used as controls. (**A**) Contour plots showing F4/80^+^ (light blue) and CD11b^+^ (grey) cells, The later population was subdivided in monocytes (Ly6C^high^Ly6G^−^ - dark blue), neutrophils (Ly6C^int^Ly6G^+^ - green) and other CD11b^+^ cells (Ly6C^−^Ly6G^−^ - red), IL-1α production was evaluated in these populations. The numbers of Ly6C^high^Ly6G^−^, Ly6C^int^Ly6G^+^ and Ly6C^−^Ly6G^−^ cells per liver, and the numbers of IL-1α^+^ cells inside each population are shown. (**B**) Immunofluorescence staining for IL-1α (green), Ly6G (red) and CD45 (gray) or IL-1α (green) and F4/80 (red) (40x magnification, scale bar corresponds to 16 μm).

The analysis of the intracellular IL-1α in CD11b^+^ and F4/80^+^ populations from the liver revealed neutrophils (CD11b^+^Ly6C^int^Ly6G^+^), but not F4/80^+^ cells, inflammatory monocytes (CD11b^+^Ly6C^high^Ly6G^−^) or other CD11b^+^ myeloid populations (CD11b^+^Ly6C^−^Ly6G^−^) as the main source of IL-1α. Although all the CD11b^+^ populations were augmented in the liver at day 7 p.i., CD11b^+^Ly6C^int^Ly6G^+^ cells were the only population that increased among the IL-1α producers (Fig. 6A). Furthermore, the immunofluorescence analysis of IL-1α-producing cells revealed a co-staining for both leukocytic marker CD45 and for the neutrophil marker Ly6G, but not for the macrophage F4/80 marker (Fig. 6B).

From these data, we can conclude that neutrophils are the inflammatory cells that act as a major source of IL-1α in the liver during blood-stage *P. chabaudi* infection.

## Discussion

Damage and inflammation in the liver are common consequences of *Plasmodium* sp. infection that contribute to the development of severe malaria^35,36^. Helping to clarify the mechanisms underlying these processes, we showed that IL-1α was mainly produced by neutrophils in the liver after blood-stage *P. chabaudi* infection and played a major role in the development of hepatic inflammation and necrosis. Corroborating our results, neutrophil infiltration and activation are associated with malaria complications in the liver, lung and brain^37–39^. Systemically, IL-1α promoted hypothermia and weight loss, but played a minor role in the control of parasitemia. These findings evidence the effect of IL-1α as an endogenous pyrogen in malaria, while the weight loss may be related to the ability of this cytokine to induce the production of TNF-α and IL-6, which are known to induce cachexia^40^. Indeed, TNF-α neutralization ameliorates hypothermia and weight loss in *P. chabaudi*-infected mice^41^.

As reported for other experimental models of hepatic injury^18,42^, the increased IL-1α production, both at the mRNA and protein levels, was observed in the liver during acute *P. chabaudi* malaria. In contrast, IL-1β remained at baseline levels, corroborating a study showing low levels of this cytokine in the serum after *P. chabaudi* infection^28^. Although hepatocytes and Kupffer cells produce IL-1α during hepatic injury^15,18,19,22,29^, a main source of this cytokine in the liver of *P. chabaudi*-infected mice was the infiltrating neutrophils. Both the immunofluorescence and flow cytometry analyses revealed IL-1α in neutrophils, but not in other liver cell populations. This observation was consistent with the independence of the NLRP3 and CASP1/11 on IL-1α release during *P. chabaudi* infection, since IL-1α secretion, mediated by NLRP3 inflammasome activation, is a particularity of macrophages and dendritic cells^23,43^. The IL-1α-producing neutrophils may be recruited into the liver during the immune response to iRBCs that accumulate in the liver vasculature^44^, and can also be attracted by the damage signals released locally during the hypoxia caused by acute malaria^36^.

Previous studies implicate IL-1α in the pathogenesis of fulminant hepatic failure and endoplasmic reticulum stress-induced liver damage^18,29^, and for the first time, we demonstrated its role in the liver injury caused by malaria. In addition to promoting necrotic lesions, IL-1α increases cellular infiltration in the liver during *P. chabaudi* infection, which corroborates the well-known role of this cytokine in inflammation^45,46^. The recruitment of IL-1α-producing neutrophils into the liver of infected mice amplifies the inflammatory response through autocrine or paracrine IL-1R signaling^21,47^ and induces the local secretion of pro-inflammatory cytokines. Thus, the low IFN-γ production by the liver CD4^+^ T cells from infected *Il1a*^−/−^ mice might be related to the ability of IL-1α to promote T cell activation, as reported during carbon tetrachloride-induced liver injury^46^. Furthermore, IL-1α signaling culminates in MyD88-mediated NFκB activation^48^, which explains the reduced TNF-α production by neutrophils, monocytes and dendritic cells and the low *Il6* mRNA levels in the liver of infected *Il1a*^−/−^ mice.

TNF-α production in the liver during malaria, which, according to our study, is potentiated by IL-1α, may be directly involved in the death of hepatocytes and the development of necrotic lesions. Free heme sensitizes hepatocytes to undergo TNF-α-mediated apoptosis during *P. chabaudi* infection, which is inhibited by the overexpression of heme oxygenase-1^36^. Furthermore, IL-1R signaling in hepatocytes also promotes TNF-α-induced cell death and neutrophil recruitment during liver inflammation^21^. Thus, we propose that the low levels of TNF-α production in the liver of infected *Il1a*^−/−^ mice attenuate the apoptosis of the hepatocytes reducing the development of secondary necrosis. Indeed, our results showing the TUNEL^+^ cells in the liver suggested that the hepatocytes died by apoptosis during acute malaria and this process was amplified by IL-1α. In addition, type I IFN and IL-12 production is also associated with induction of liver injury during experimental malaria^36,49^ as well as the MyD88 adaptor, which is shared by the TLR and IL-1R signaling pathways^49^.

In summary, we showed that *P. chabaudi* infection induces the recruitment of IL-1α-producing neutrophils into the liver. IL-1α, released locally, promotes the inflammatory response and, in particular, the production of TNF-α, which has been directly implicated in the apoptosis of hepatocytes and, as a consequence, liver tissue necrosis occurs. IL-1α also contributes to the clinical manifestations of acute malaria, such as changes in body temperature and weight loss. This study helps to characterize the pathophysiology of liver disease during malaria and opens future perspectives for new therapeutic approaches.

## Methods

### Mice, parasite and infection

Six- to eight-week-old C57BL/6 (Jackson Laboratory, USA), *Il1a*^−/−^ (Jackson Laboratory), *Nlrp3*^−/−^ (Genentech, USA) and *Casp1/11*^−/−^ (Jackson Laboratory) male mice were housed under specific pathogen-free conditions at the Isogenic Mice Facility (ICB-USP, Brazil). The *P. chabaudi* AS parasites were maintained as described previously^50^. The mice were infected with 1 × 10^6^ infected red blood cells by the intraperitoneal route (iRBCs).

### Parasitemias and analysis of body weight and temperature

The parasitemias were assessed by a microscopic examination of Giemsa-stained thin tail blood smears. The body weight change was calculated in relation to the day 0 weight and the mice were weighted using an analytical balance (Sartorious, USA). The body temperature was measured by using a digital thermometer (Kent Scientific Co., USA).

### Ethics statement

All of the experimental procedures were in accordance with the national regulations of the ethical guidelines for mice experimentation and welfare of the Council for Control of Animal Experimentation (CONCEA: Conselho Nacional de Controle de Experimentação Animal) - Brazil, and the protocols were approved by the Animal Health Committee of the Biomedical Sciences Institute of the University of São Paulo, with permit numbers 175/2011 and 29/2016.

### Histological analysis

The liver sections were fixed in formalin for 24 h at room temperature. The fixed organs were embedded in paraffin, sectioned (5 μm) and stained with hematoxylin and eosin (H&E). The histological analysis was performed by a pathologist blinded to the experimental groups on a Leica DM2000 microscope coupled to a Leica MC170 camera (Germany). The liver lesions were scored for multiple parameters (adapted from^51^), including (i) inflammatory cell infiltration (cell type, distribution and severity) and (ii) cellular changes (character - e.g. necrosis, lipidosis, hypertrophy, distribution and severity), and the severity score ranged from 0 to 4 (0, absent; 1, minimal; 2, mild; 3, moderate; and 4, marked), as shown in Table 1.

**Table 1:** Grading scheme for the liver lesions.

Severity	Proportion of liver affected	Score	Quantifiable finding
Minimal	Very small amount	1	1–2 foci
Mild or slight/few	Small amount	2	3–6 foci
Moderate or several	Medium amount	3	7–12 foci
Marked or many	Large amount	4	>12 foci, coalescing

### Immunofluorescence

The livers were fixed in 4% paraformaldehyde for 12 hours. The tissue sections (5 μm thick) were cut using a CM3050S Cryostat (Leica). The slices were permeabilized and blocked with 0.3% Triton-X plus 1% BSA for 1 hour at room temperature. The slides were then stained with a FITC-labeled anti-IL-1α monoclonal antibody (mAb; ALF-161), a PE-labeled anti-Ly6G mAb (IA8) and an APC-labeled anti-CD45 mAb (30-F11) for 4 hours at 4 °C. All the antibodies were purchased from eBioscience (Thermo Fisher, USA). After wash step, the slides were stained for 30 min with rhodamine-phalloidin (Thermo Fisher), and mounted with Fluoromount-G containing 4′,6-diamidino-2-phenylindole (DAPI; Southern Biotechnologies, USA). The sections were visualized and analyzed by confocal microscopy (Zeiss LSM 780, Germany).

### Serum biochemical measurements

Levels of alanine aminotransferase (ALT) and aspartate aminotransferase (AST) were measured in the plasma using the Catalyst One Biochemical Analyzer (Idexx Laboratories, USA). The levels of lactate dehydrogenase (LDH) in the plasma were measured in a Beckman Coulter AU 680 device (USA).

### Cell suspensions

The liver and spleen cells were maintained in cold RPMI 1640 supplemented with 3% heat-inactivated fetal calf serum, L-glutamine (2 mM), sodium pyruvate (1 mM), 2-mercaptoethanol (50 μM), penicillin (100 U/ml) and streptomycin (100 μg/ml). All the supplements were purchased from Life Technologies (USA). After PBS perfusion, the liver was removed, treated with 0.04% type IV collagenase (Sigma-Aldrich) and macerated with a cell strainer (Corning, USA). To eliminate the RBCs, spleen and liver cells were incubated in lysis buffer (40 mM NH_4_Cl and 4.2 mM Tris pH 7.4) for 5 min at 4 °C. After applying the liver cell suspension to a 35% Percoll (GE Healthcare Life Sciences, England) gradient centrifugated for 20 minutes at 500 g and room temperature, the leukocytes were obtained from the pellet.

### Cell phenotyping

The spleen and liver cells (1 × 10^6^) were stained with PE-, FITC-, PerCP-, APC-, APC Cy7-, PECy7-, Pacific Blue-, or AmCyan-labeled mAbs (BD Pharmingen and eBioscience) to CD45 (30-F11), CD4 (GK1.5), CD8 (53–6.7), NK1.1 (PK136), F4/80 (BM8), CD11b (M1/70), CD11c (HL3), Gr1 (RBC-8C5), Ly6C (HK1.4), and Ly6G (1A8). Next, the cells were stained for viability using a Live/Dead kit (Life Technologies) and analyzed by flow cytometry using a FACSCanto device with DIVA software (BD Biosciences). The data were analyzed with FlowJo software v.7.2.2 (Tree Star Inc., USA).

### Cytokine detection

The IL-1α and IL-1β quantification in the liver macerate supernatants was determined by ELISA (BD Biosciences). For intracellular IL-1α, IFN-γ and TNF-α detection, the spleen and liver cells (1 × 10^6^) were incubated with GolgiPlug or GolgiStop (BD Biosciences) for 6 h at 37 °C in 5% CO_2_. After washing, the cells were surface stained with the previous described mAbs. The cells were then fixed with Cytofix/Cytoperm buffer, stained with FITC-labeled mAb to IL-1α (eBioscience) and PE-labeled mAb to IFN-γ or TNF-α (BD Pharmingen) and analyzed by flow cytometry. All the reagents were obtained from BD Pharmingen. The stained cells were analyzed by flow cytometry using a FACSCanto device with DIVA software.

### Real-time polymerase chain reaction (RT-PCR)

The RNA samples were isolated from the total liver tissue using an RNeasy Mini Kit (Qiagen, USA). The reverse transcription of the RNA was performed using a High Capacity cDNA Reverse Transcription Kit (Applied Biosystems, USA). The resulting cDNAs were amplified by PCR with the Maxima SYBR Green/ROX qPCR Master Mix (Thermo Fisher) in a QuantStudio 3 Real Time PCR System (Applied Biosystems). The gene expression was calculated using the 2^(−ΔΔCT)^ relative quantification method. The primers used for the RT-PCR were as follows: for *Il1a*, ACCTGCAGTCCATAACCCA (forward) and GACAAACTTCTGCCTGACGA (reverse); for *Tnfa*, CATCTTCTCAAAATTCGAGTGACAA (forward) and TGGGAGTAGACAAGGTACAACCC (reverse); for *Gapdh*, TGAAGCAGGCATCTGAGGG (forward) and CGAAGGTGGAAGAGTGCGAG (reverse); for *Il6*, ATGGATGCTACCAAACTGGAT (forward) and TGAAGGACTCTGGCTTTGTCT (reverse); and for *Ifng*, TCAAGTGGCATAGATGTGGAAGAA (forward) and TGGCTCTGCAGGATTTTCATG (reverse).

### TUNEL assay

The pieces of liver tissue were frozen in the presence of Tissue-Tek O.C.T. (Sakura Finetek, USA). *In situ* detection of DNA fragmentation in 5-μm-thick slices was performed using he DeadEnd Fluorometric TUNEL System (Promega, USA).

### Statistical analysis

The statistical analyses were performed with the Kruskal-Wallis, Mann-Whitney and Two-way ANOVA with Sidak’s multiple comparison tests, as described in each figure, using the GraphPad Prism 6 software.

## Supplementary information

Supplementary information

## Data Availability

The authors declare that the data will be available without restrictions.
