# Supplementary material for: IL-1α promotes liver inflammation and necrosis during blood-stage Plasmodium chabaudi malaria
Source: Sci Rep. 2019 May 20;9:7575. doi: 10.1038/s41598-019-44125-2 (PMC6527574; doi:10.1038/s41598-019-44125-2)
Supplement: Supplementary file 1 — Supplementary information [file 41598_2019_44125_MOESM1_ESM.pdf]

IL-1α promotes liver inflammation and necrosis during blood-stage *Plasmodium chabaudi* malaria

Maria Nogueira de Menezes[1,*], Érika Machado Salles[1], Flávia Vieira[1], Eduardo Pinheiro Amaral[1], Vanessa Zuzarte-Luís[2], Alexandra Cassado[1], Sabrina Epiphanio[3], José Alvarez[1], José Carlos Alves-Filho[4], Maria Manuel Mota[2], Maria Regina D'Império-Lima[1,*]

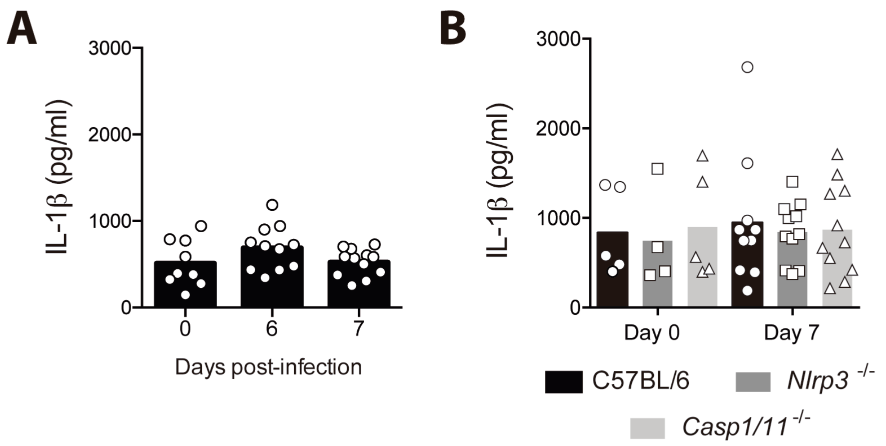

Supplementary Fig. S1. IL-1β production is not increased in the liver during acute *P. chabaudi* malaria. The mice were analyzed at days 6 and 7 p.i. with 1 x 10^6 *P. chabaudi*-iRBCs. Non-infected mice (day 0) were used as controls. (A) The IL-1β levels in the liver cell supernatants from C57BL/6 mice. (B) The IL-1β levels in the liver cell supernatants from C57BL/6, *Nlrp3*-/- and *Casp1/11*-/- mice. The data were pooled from three independent experiments (*n* = 4-11). No significant differences were observed between the mouse groups, using the Kruskal-Wallis test.

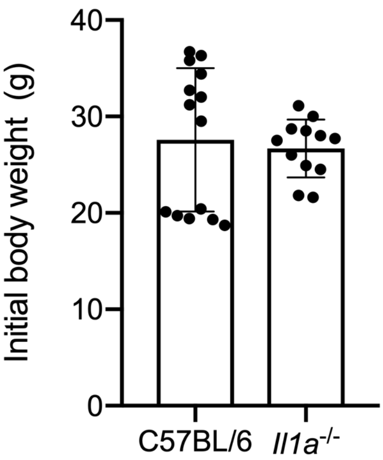

Supplementary Fig. 2. C57BL/6 and *Il1a*$^{-/-}$ mice presented similar initial body weights. Body weights were determined shortly before *P. chabaudi* infection. The data were pooled from three independent experiments (*n* = 12-14). No significant difference was observed between the mouse groups, using the Mann-Whitney test.

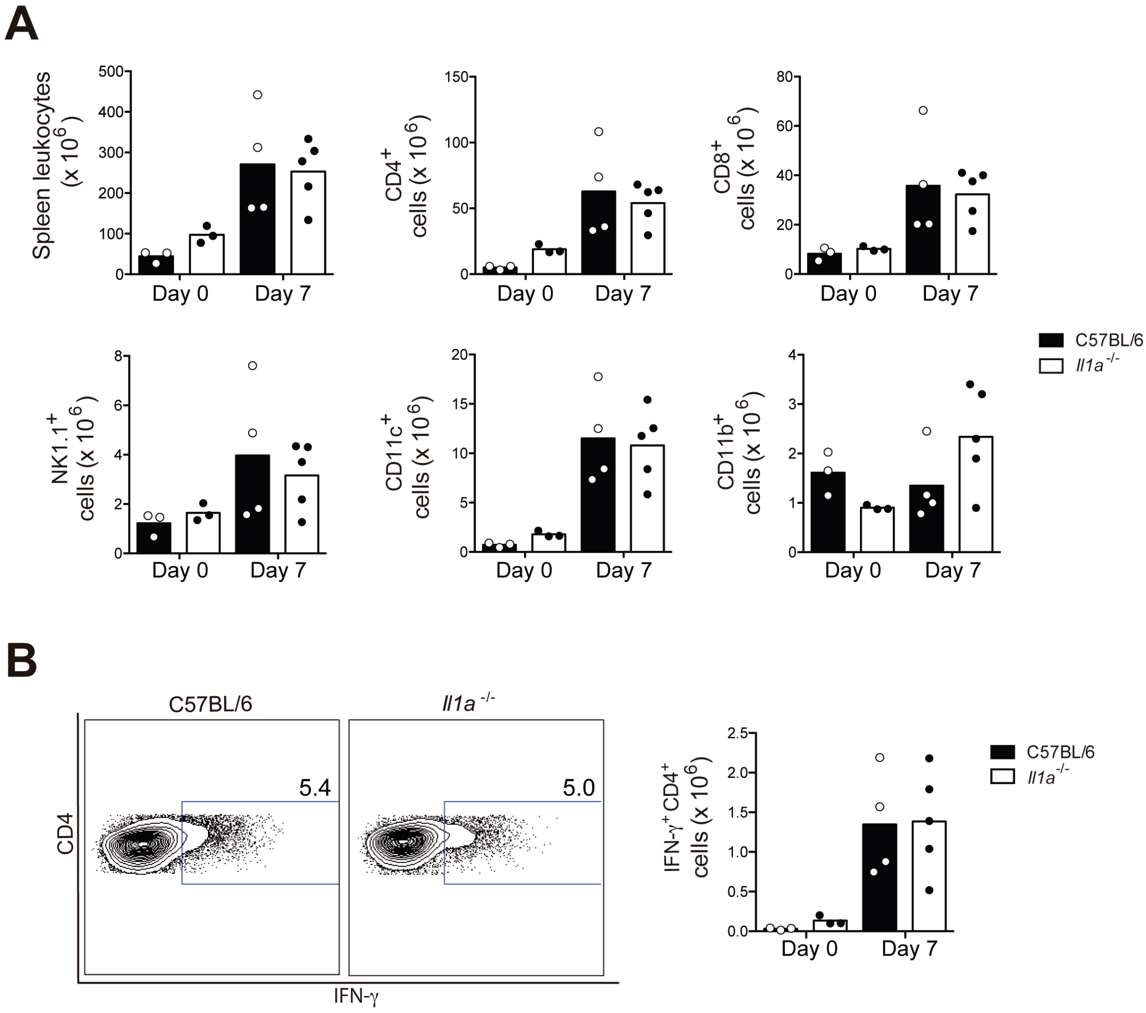

Supplementary Fig. 3. Leukocyte populations and IFN-γ production by CD4^{+} cells are similarly increased in the spleen of *P. chabaudi*-infected C57BL/6 and *Il1a*$^{-/-}$ mice. C57BL/6 and *Il1a*$^{-/-}$ mice were analyzed at day 7 p.i. with 1 x 10^6 *P. chabaudi*-iRBCs. Non-infected mice (day 0) were used as controls. (A) Total number of leukocytes and CD4^{+}, CD8^{+}, NK1.1^{+}, CD11c^{+} and CD11b^{+} cells per spleen. (B) Contour plots showing intracellular IFN-γ production in CD4^{+} cells and the IFN-γ^{+}CD4^{+} cell numbers per spleen. The data are expressed as the means ± SD (*n* = 3-5) of one representative experiment out of three. No significant difference was observed between the C57BL/6 and *Il1a*$^{-/-}$ mouse groups, using the Mann-Whitney test.
